# Multigenic engineering of the chloroplast genome in the green alga *Chlamydomonas reinhardtii*

**DOI:** 10.1099/mic.0.000910

**Published:** 2020-04-06

**Authors:** Marco Larrea-Alvarez, Saul Purton

**Affiliations:** ^1^​ Algal Research Group, Institute of Structural and Molecular Biology, University College London, Gower Street, London, WC1E 6BT, UK; ^†^​ Present address: School of Biological Sciences and Engineering. Yachay-Tech University Hacienda San José, Urcuquí-Imbabura, Ecuador

**Keywords:** *Chlamydomonas*, chloroplast, genetic engineering, microalgae, plastome

## Abstract

The chloroplast of microalgae such as *Chlamydomonas reinhardtii* represents an attractive chassis for light-driven production of novel recombinant proteins and metabolites. Methods for the introduction and expression of transgenes in the chloroplast genome (=plastome) of *C. reinhardtii* are well-established and over 100 different proteins have been successfully produced. However, in almost all reported cases the complexity of the genetic engineering is low, and typically involves introduction into the plastome of just a single transgene together with a selectable marker. In order to exploit fully the potential of the algal chassis it is necessary to establish methods for multigenic engineering in which many transgenes can be stably incorporated into the plastome. This would allow the synthesis of multi-subunit proteins and the introduction into the chloroplast of whole new metabolic pathways. In this short communication we report a proof-of-concept study involving both a combinatorial and serial approach, with the goal of synthesizing five different test proteins in the *C. reinhardtii* chloroplast. Analysis of the various transgenic lines confirmed the successful integration of the transgenes and accumulation of the gene products. However, the work also highlights an issue of genetic instability when using the same untranslated region for each of the transgenes. Our findings therefore help to define appropriate strategies for robust multigenic engineering of the algal chloroplast.

Current commercial production of recombinant molecules such as therapeutic proteins and bioactive metabolites relies almost exclusively on heterotrophic cell platforms such as bacteria, yeasts and mammalian cell lines [1]. However, there is increasing interest in the exploitation of eukaryotic microalgae as low-cost, phototrophic platforms [2, 3]. Green algal species such as *Chlamydomonas reinhardtii*, *Chlorella vulgaris* and *Haematococcus pluvialis* can be cultivated at scale in simple photobioreactor systems using a basic growth medium [4, 5]. Furthermore, these species have GRAS status and do not harbor harmful endotoxins or viral and prion contaminants, thereby simplifying the downstream processing steps [6]. In addition, plants and algae possess a unique biosynthetic and storage organelle – the chloroplast – that is not present in animal or fungal cells, and which is the site of synthesis of key metabolites such as fatty acids, terpenoids, carbohydrates and tetrapyrroles, as well as the major proteins of the photosynthetic apparatus [7]. As the chloroplast contains its own minimal genome (=plastome) and genetic system that is derived from its cyanobacterial ancestor [8], this organelle represents an attractive sub-cellular ‘chassis’ on to which can be bolted new metabolic pathways through chloroplast genetic engineering [9, 10].

DNA transformation of the algal chloroplast has been reported for a handful of species, but the technology is most advanced for *C. reinhardtii* [11]. Numerous studies have shown that insertion of foreign DNA into the plastome occurs exclusively via homologous recombination allowing precise and predictive targeting of transgenes into specific loci [12]. High-level expression of the transgenes is achieved by fusing them to *cis* elements (promoters and untranslated regions) of highly expressed chloroplast genes, and transgene expression is stable in the absence of selection since there are no gene silencing mechanisms in the chloroplast. Methods have been developed for generating marker-free transgenic lines [13] and for regulated expression of the transgenes [9], together with a codon-reassignment method that ensures their biocontainment [14]. In addition, we are now starting to see the application of synthetic biology strategies for the rapid and standardised assembly of expression cassettes [3].

Our survey of the literature has revealed that over 100 different foreign proteins have been produced successfully in the *C. reinhardtii* chloroplast. These include a wide range of therapeutic proteins such as vaccines, hormones and antibodies [11]; industrial enzymes [15, 16], and enzymes for synthesizing novel metabolites in the organelle [17–21]. However, in almost all cases the genetic engineering has involved the insertion of just one transgene (or a single transgene together with a bacterial gene such as *aadA* or *aphA-6* as the selectable marker [13]). In the few remaining reports, two transgenes have been employed – either as a dicistronic operon [22]; as two linked gene cassettes inserted into one site of the plastome [18], or as independent gene cassettes introduced by co-transformation into two separate loci [23]. In order to fully exploit the algal chloroplast as an expression platform there is a need to advance the genetic engineering technology such that multiple transgenes can be introduced into the plastome. This would allow the biosynthesis of multi-subunit complexes such as the bacterial nitrogenase enzyme (a long-standing goal in chloroplast engineering [24]) and elaborate metabolic engineering involving multiple enzyme steps.

In this study we have explored multigenic engineering by combining two approaches: namely, the integration of three gene cassettes into a single neutral locus, followed by a further round of transformation to integrate a fourth gene cassette plus the *aadA* marker cassette at a second locus. The four genes were selected from previous studies in our group where each coding sequence was codon-optimised for expression in the chloroplast, and each was modified to encode the haemagglutinin epitope tag (HA-tag), YPYDVPDYA at the C terminus [25]. The gene products are unrelated, and were chosen based on their different sizes and the different levels of accumulation observed previously in chloroplast transformants expressing just the single gene. The four genes are: (i) *splB* encoding a serine protease from the bacterium *Staphylococcus aureus* that cleaves at the recognition sequence W-E-l-Q-↓-X [26]. SplB accumulates to a high level in the chloroplast (>1 % total soluble protein) and has application as a highly specific enzyme for *in vivo* processing of recombinant proteins [27]; (ii) *codA* encoding a variant of *E. coli* cystosine deaminase that can be exploited as a negative marker in the *C. reinhardtii* chloroplast catalyzing the conversion of 5-fluorocytosine into the toxic 5-fluorouracil [25]; (iii) *ibv-ctb*, designed to encode a chimeric protein that could serve as an oral vaccine for the poultry pathogen, infectious bronchitis virus (IBV). The protein comprises three antigenic sections of the viral N protein [28] fused to the β-subunit of cholera toxin (CTB), which can serve as an immunological adjuvant [11]; (iv) *cpl-1* encoding a bacteriophage endolysin that targets the bacterial pathogen *Streptococcus pneumoniae* and has been shown to be active when synthesized in the *C. reinhardtii* chloroplast [29].

As shown in Fig. 1a, the first three genes were assembled into a single plasmid (pICS) that is based on the chloroplast expression vector pASapI [30]. Details of this vector and the cloning strategy used are given in the supplementary data (available in the online version of this article). A key feature of pASapI is that it carries a wild–type copy of the essential photosystem II gene, *psbH* on the right homology arm. Consequently, *psbH* can be used as a selectable marker for phototrophic restoration of Δ*psbH* strains such as TN72 in which *psbH* has been deleted using the *aadA* cassette. Transformant colonies are selected on minimal medium and scored for loss of the spectinomycin resistance conferred by *aadA* [30]. When building each of the cassettes, the three coding sequences were fused to a promoter/5′UTR element from a different endogenous gene (namely, *psaA-1*, *petB* and *atpA*) in order to minimise the competition for *trans*-acting factors required for mRNA stability and translation initiation that bind to the 5′UTR [31]. Conversely, the same 3′UTR element from *rbcL* was used in each case since the choice of 3′UTR has little effect on the level of transgene expression [32]. However, it is important to note (as discussed below) that the *codA* cassette used in the making of pICS contained a newer and shorter version of the *rbcL* 3′UTR element (i.e. 258 bp versus 407 bp) in which unnecessary sequence spanning the end of the *rbcL* coding region was removed [25].

**Fig. 1.**
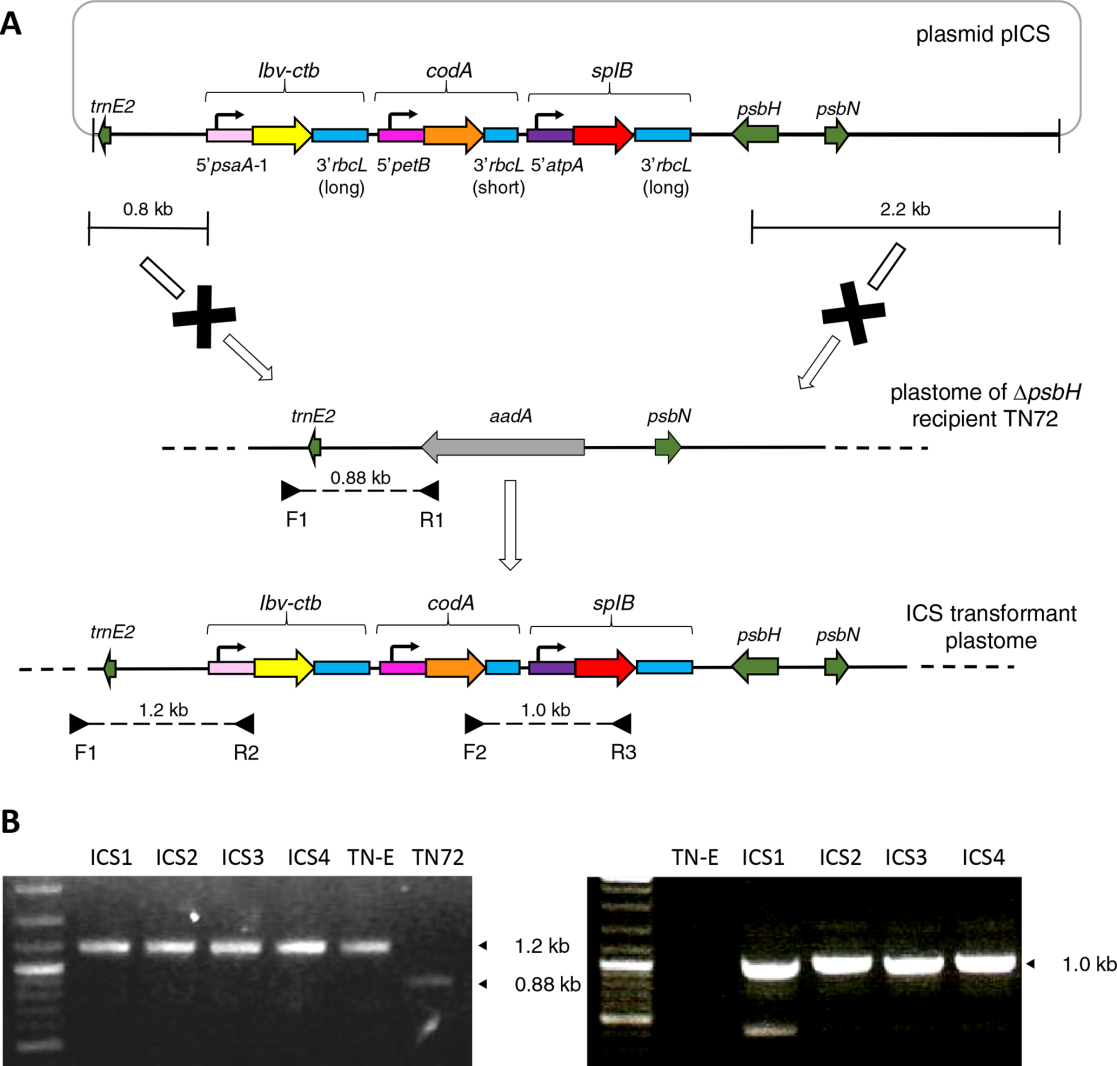
Introduction of three gene cassettes into the *C. reinhardtii* plastome using a combinatorial approach. Design of the cassettes, chloroplast transformation and analysis of transformant lines were carried out using the methods described previously [24, 30]. (a) The transformation plasmid pICS is derived from the expression vector pASapI and was assembled using three transgene cassettes in which each coding sequence (*ibv-ctb*, *codA* and *splB*) was codon-optimised and extended to encode a C-terminal HA tag. Each was fused to a promoter/5′UTR element from a different endogenous gene. The same *rbcL* 3′UTR was used for all cassettes although in the case of *codA* a shorter version of the 3′UTR was used. The cassette cluster is flanked by homology arms with the right-hand arm carrying a copy of *psbH*. This allows rescue of a *psbH* knockout mutant (TN72) to phototrophy, and targeting of the cluster to an intergenic locus on the plastome downstream of *psbH*. (b) Confirmation of integration of the cluster in four transformant lines (ICS1–4) by colony PCR and agarose gel analysis. Strain TN-E was generated using the ‘empty’ pASapI and serves as a negative control. The left-hand figure shows the result of a three-primer reaction (primers F1, R1 and R2) in which a novel 1.2 kb band is obtained from transgenic copies of the polyploid plastome, whereas a 0.88 kb band arises from the TN72 plastome. The absence of this band in the transformant lines is indicative of homoplasmy. Integration of the cluster was further confirmed using internal primers (F2 and R3) as shown in the right-hand figure.

Four phototrophic colonies (ICS1 – ICS4) were selected following glass-bead mediated transformation of TN72 [33] with plasmid pICS, and were restreaked twice to single colonies to obtain homoplasmic transformants. As shown in Fig. 1b, PCR analysis confirmed the integration of the three-cassette cluster into the plastome. The four transformant lines were then subjected to Western blot analysis in which cell lysates were fractionated by SDS polyacrylamide gel electrophoresis and probed using an antibody against the HA epitope present on each of the three recombinant proteins (Fig. 2). Comparison of the four lines to control lines containing just one of the transgene cassettes or an empty cassette lacking any coding sequence (strain rTN72) confirmed that all four are producing the three recombinant proteins, with high levels of CodA and SplB and a much lower level of IBV-CTB. This low level probably reflects a higher rate of protein turnover rather than a lower rate of expression, since the *psaA-1* element used to drive *ibv-ctb* expression is actually stronger than the *atpA* element driving *splB* expression [30]. The artificial and composite structure of IBV-CTB (three epitope regions from the N protein together with the CTB protein fused into a single polypeptide) probably limits folding into a stable, protease-resistant form, unlike CodA and SplB which are two natural bacterial enzymes. The observed reduction in RbcL levels in the ICS transformants compared to the single transformants (Fig. 2) is possibly due to the presence of multiple copies of the *rbcL* 3′UTR resulting in the titrating out of *trans*-acting factors required for 3′ processing and stability of the *rbcL* transcript [34]. This reduced level is also reflected in a small reduction in growth performance of the ICS transformant compared to the rTN72 control (Fig. S1).

**Fig. 2.**
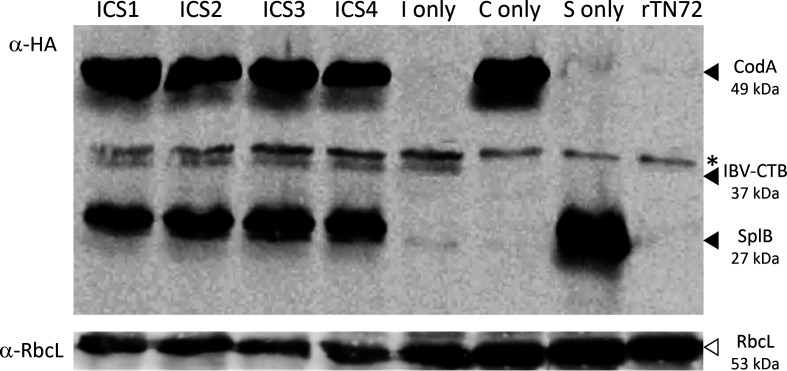
Western blot analysis demonstrates accumulation of the three recombinant proteins. Cell extracts of the four multigenic lines (ICS1–4) were analysed using primary antibodies to the HA epitope tag on each recombinant protein as described [25]. Antibodies to the chloroplast protein RbcL were used as a loading control. For comparison, a negative control transformant (rTN72) and three ‘single transgene’ transformant lines of TN72 were included in the analysis (labelled ‘I only’, *etc*.). The recombinant proteins and their predicted molecular weights are indicated together with an unknown endogenous protein (*) that is detected by the α-HA antibodies.

Having demonstrated that three foreign proteins could be synthesized in a single transgenic line, we then sought to further engineer this line by addition of two more transgenes at a second locus. We therefore built a second transformation plasmid (named pAP) that was designed to target the *aadA* cassette that confers spectinomycin-resistance [35] and a cassette for expression of the endolysin gene *cpl-1* [29] into the middle of the non-essential chloroplast gene, *chlL*. This gene encodes a subunit of the dark-operative protochlorophyllide reductase and a knockout of *chlL* gives rise to a ‘yellow-in-the-dark’ phenotype [36]. Consequently, the insertion of transgenes into *chlL* can be used as a simple screen to distinguish genuine *aadA* transformants from spectinomycin-resistant clones arising from spontaneous mutation [13].

The pAP plasmid was used to transform line ICS3 to spectinomycin resistance as previously described [33]. Three colonies (ICS3-AP #1–3) were selected; confirmed to be transformants by their yellow-in-the-dark phenotype, and checked for the correct insertion of the two gene cassettes by similar PCR analysis to that carried out in Fig. 1. Since the *cpl-1* gene was designed to encode an HA-tagged version of Cpl-1 [29], we anticipated that Western analysis of the ICS3-AP transformants using the anti-HA antibodies would demonstrate the synthesis of four different recombinant proteins: namely, Cpl-1, together with IBV-CTB, CodA and SplB. However, only Cpl-1 and IBV-CTB could be detected in the three transformant lines despite CodA and SplB being readily detectable in the ICS3 recipient line (Fig. 3b). We therefore reasoned that the cluster of three cassettes was genetically unstable, and that selection and re-streaking of the *aadA* transformants resulted in the loss or rearrangement of the *codA* and *splB* cassettes. This hypothesis was confirmed by PCR analysis and sequencing of the PCR products, which showed that homologous recombination between the two longer copies of the *rbcL* 3′UTR within the *ibv-ctb* and *splB* cassettes had resulted in the loss of both *codA* and *splB* (Fig. 3a). As seen in Fig. 3c, PCR analysis with primer F2 and R5 gave rise to a 1.0 kb band rather than the 5.0 kb band expected for the intact gene cluster. Furthermore, no 2.4 kb band was seen when an alternative reverse primer (R4) from within the *splB* gene was used with F2, suggesting that the transformants were homoplasmic with all plastome copies carrying the *codA-splB* deletion. For the ICS3 recipient line, the 2.4 kb band was obtained but so was the 1.0 kb band. This indicates that recombination between the *rbcL* direct repeats is occurring in the ICS3 strain such that a heteroplasmic population of plastomes exists, and that the selection for individual transformants drives this to a homoplasmic state since there is no counter-selection to retain the intact cluster. Interestingly, despite the reduced ploidy of the intact cluster in ICS3 due to this heteroplasmicity, we still achieve levels of SplB and CodA accumulation in ICS3 (and the other ICS lines) that are approximately 50–90 % of that seen for the homoplasmic transformant lines ‘C only’ and ‘S only’ (Fig. 2). This observation supports the findings of Eberhard *et al*. [37] that chloroplast protein synthesis is relatively insensitive to changes in gene copy number, and a marked reduction of plastome ploidy has only a small effect on protein synthesis rates. Finally, our PCR analysis did not reveal any evidence of recombination between either of the two longer (407 bp) copies of the *rbcL* 3′UTR and the shorter (258 bp) copy on *codA*. This suggests that there is a lower size limit, somewhere between 0.25 kb and 0.4 kb, for efficient intramolecular homologous recombination in the *C. reinhardtii* chloroplast. This range correlates well with an earlier study of different sized direct repeats in the algal plastome where Fischer *et al.* [38] found that a 230 bp repeat did not give rise to recombination whereas repeats of 483 bp and 832 bp did.

**Fig. 3.**
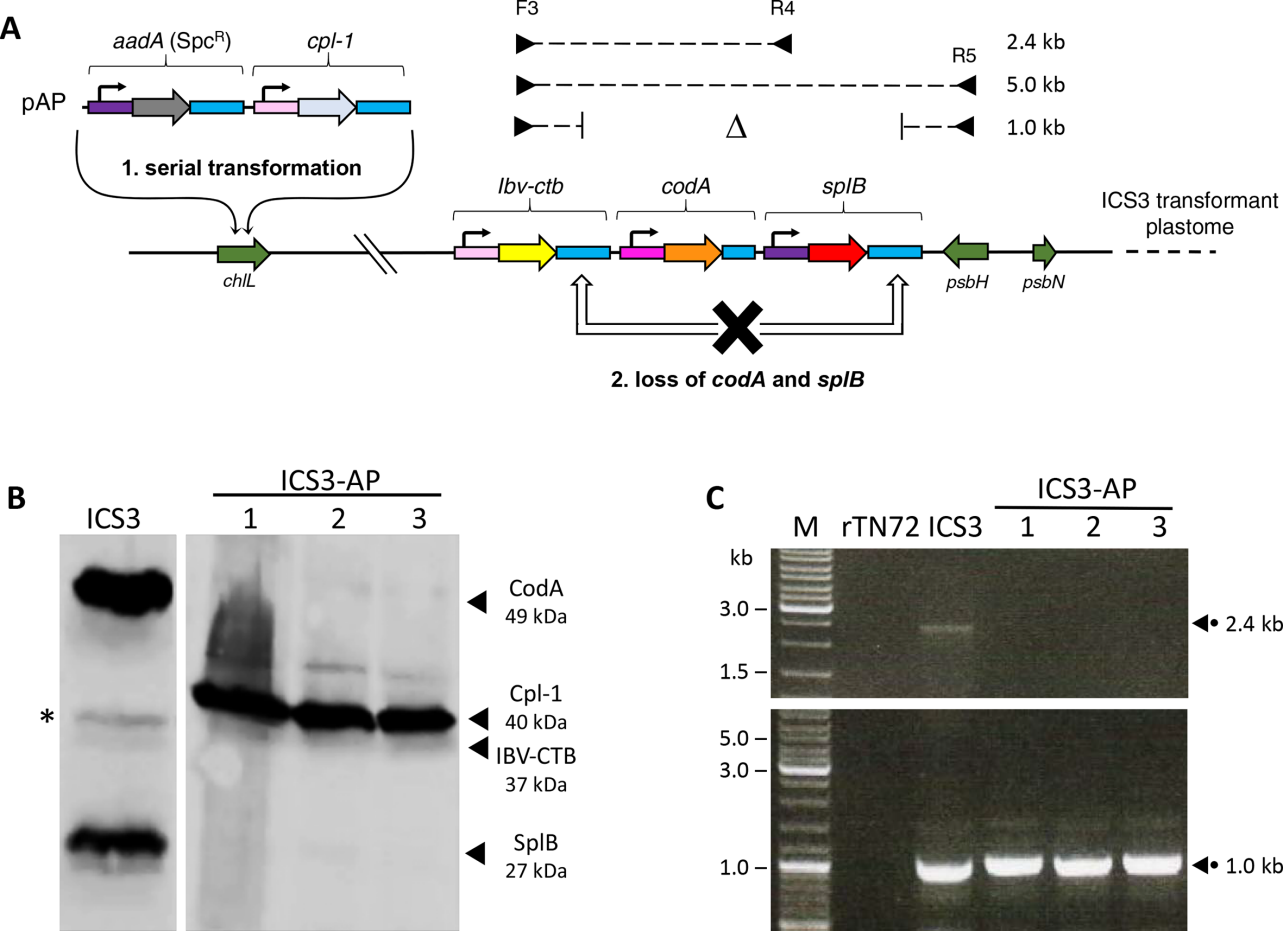
Serial transformation reveals genetic instability of the original gene cassette cluster. (a) The transformant line ICS3 was used as a recipient for a further round of transformation in which two transgene cassettes was targeted to a second plastome locus within the non-essential gene *chlL*,~55 kb from the *psbH* locus. The two cassettes were *aadA* (encoding a bacterial enzyme conferring resistance to spectinomycin [35]) and *cpl-1* (encoding an HA-tagged ‘phage endolysin [29]). The gene cassettes were cloned into a transformation plasmid with appropriate homology arms (plasmid pAP). Homoplasmic transformant lines were generated exactly as for the pICS transformants above, except that selection was for spectinomycin resistance [33]. (b) Western blot analysis of three transformant lines (ICS3-AP1, 2 and 3) confirms the expression of the 40 kDa Cpl-1, together with the 37 kDa IBV-CTB from the recipient ICS3 strain, but the CodA and SplB proteins are no longer detectable. (c) PCR analysis confirms that the *codA* and *splB* cassettes have been lost from the three transformants through intramolecular recombination between the directly repeated copies of the *rbcL* 3′UTR as shown by the 1.0 kb band. This instability is seen in the ICS3 line itself with the presence of the 1.0 kb band, in addition to the 2.4 kb band from the intact cassette cluster.

In conclusion, this short report demonstrates that multiple transgene cassettes can be inserted into the *C. reinhardtii* plastome to allow ever-more complex genetic engineering such as that required for introduction of novel metabolic pathways. A cluster of cassettes can be targeted to a chosen locus in a single transformation event, and then further clusters can be introduced into additional loci by serial transformation. However, a clear caveat is that the endogenous *cis* elements (promoters, 5′UTRs and 3′UTRs) used to drive expression of the transgenes should not be used multiple times, and/or should be smaller than ~0.2 kb to limit recombination between each other and between the endogenous gene itself. Alternately, heterologous elements from other green algae or from cyanobacteria could be used where the primary DNA sequence is sufficiently different to prevent recombination. Several studies have demonstrated that heterologous promoters and 3′UTRs are functional in the algal chloroplast, whereas 5′UTRs are much more species-specific [39, 40]. Nevertheless, directed mutagenesis of *C. reinhardtii* 5′UTRs could be employed as an alternative strategy to both reduce their homology and improve their performance [41]. With these considerations in mind, we are currently developing a DNA parts library for rapid and standardized assembly of transgene cassettes based on the Start-Stop Assembly method described recently by Taylor *et al.* [42].

## Supplementary Data

Supplementary material 1
