# Supplementary material for: Multigenic engineering of the chloroplast genome in the green alga Chlamydomonas reinhardtii
Source: Microbiology (Reading). 2020 Apr 6;166(6):510–5. doi: 10.1099/mic.0.000910 (PMC7376270; doi:10.1099/mic.0.000910)
Supplement: Supplementary material 1 [file mic-166-510-s001.pdf]

Supplementary Data

Multigenic engineering of the chloroplast genome in the green alga *Chlamydomonas reinhardtii*

Marco Larrea-Alvarez and Saul Purton

1. pASapI map and DNA sequence

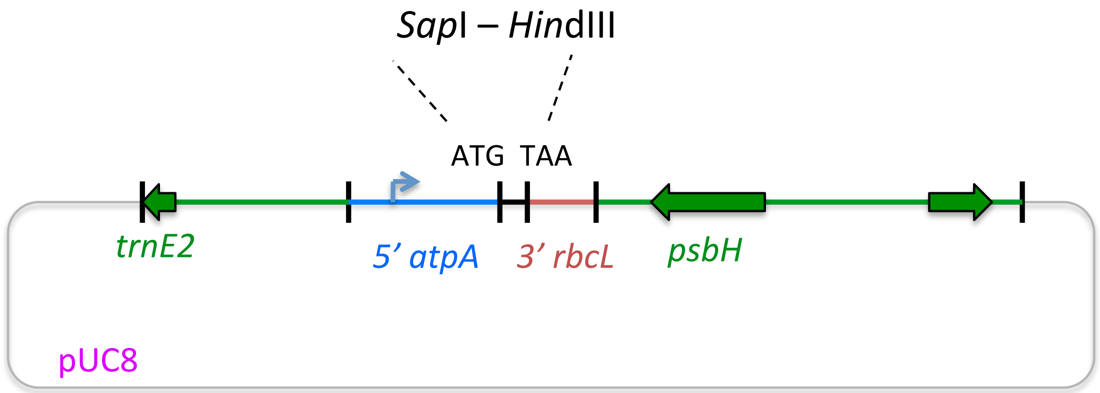


```
> pASapI chloroplast expression vector - 6622 bp
agcttggcactggccgtcgttttacaacgtcgtgactgggaaaaccctggcgttacccaacttaatcgccttgcagcacatccc
cctttcgccagctggcgtaatagcgaagaggcccgcaccgatcgcccttcccaacagttgcgcagcctgaatggcgaatggcgc
ctgatgcggtattttctccttacgcatctgtgcggtatttcacaccgcatatggtgcactctcagtacaatctgctctgatgcc
gcatagttaagccagccccgacacccgccaacacccgctgacgcgccctgacgggcttgtctgctcccggcatccgcttacaga
caagctgtgaccgtctccgggagctgcatgtgtcagaggttttcaccgtcatcaccgaaacgcgcgagacgaaagggcctcgtg
atacgcctatttttataggttaatgtcatgataataatggtttcttagacgtcaggtggcacttttcggggaaatgtgcgcgga
acccctatttgtttatttttctaaatacattcaaatatgtatccgctcatgagacaataaccctgataaatgcttcaataatat
tgaaaaaggaagagtatgagtattcaacatttccgtgtcgcccttattcccttttttgcggcattttgccttcctgtttttgct
cacccagaaacgctggtgaaagtaaaagatgctgaagatcagttgggtgcacgagtgggttacatcgaactggatctcaacagc
ggtaagatccttgagagttttcgccccgaagaacgttttccaatgatgagcacttttaaagttctgctatgtggcgcggtatta
tcccgtattgacgccgggcaagagcaactcggtcgccgcatacactattctcagaatgacttggttgagtactcaccagtcaca
gaaaagcatcttacggatggcatgacagtaagagaattatgcagtgctgccataaccatgagtgataacactgcggccaactta
cttctgacaacgatcggaggaccgaaggagctaaccgcttttttgcacaacatgggggatcatgtaactcgccttgatcgttgg
gaaccggagctgaatgaagccataccaaacgacgagcgtgacaccacgatgcctgtagcaatggcaacaacgttgcgcaaacta
ttaactggcgaactacttactctagcttcccggcaacaattaatagactggatggaggcggataaagttgcaggaccacttctg
cgctcggcccttccggctggctggtttattgctgataaatctggagccggtgagcgtgggtctcgcggtatcattgcagcactg
gggccagatggtaagccctcccgtatcgtagttatctacacgacggggagtcaggcaactatggatgaacgaaatagacagatc
gctgagataggtgcctcactgattaagcattggtaactgtcagaccaagtttactcatatatactttagattgatttaaaactt
catttttaatttaaaaggatctaggtgaagatcctttttgataatctcatgaccaaaatcccttaacgtgagttttcgttccac
tgagcgtcagaccccgtagaaaagatcaaaggatcttcttgagatcctttttttctgcgcgtaatctgctgcttgcaaacaaaa
aaaccaccgctaccagcggtggtttgtttgccggatcaagagctaccaactctttttccgaaggtaactggcttcagcagagcg
cagataccaaatactgtccttctagtgtagccgtagttaggccaccacttcaagaactctgtagcaccgcctacatacctcgct
ctgctaatcctgttaccagtggctgctgccagtggcgataagtcgtgtcttaccgggttggactcaagacgatagttaccggat
aaggcgcagcggtcgggctgaacggggggttcgtgcacacagcccagcttggagcgaacgacctacaccgaactgagatacctа
cagcgtgagctatgagaaagcgccacgcttcccgaagggagaaaggcggacaggtatccggtaagcggcagggtcggaacagga
gagcgcacgagggagcttccagggggaaacgcctggtatctttatagtcctgtcgggtttcgccacctctgacttgagcgtcga
tttttgtgatgctcgtcaggggggcggagcctatggaaaaacgccagcaacgcggcctttttacggttcctggccttttgctgg
ccttttgctcacatgttctttcctgcgttatcccctgattctgtggataaccgtattaccgcctttgagtgagctgataccgct
cgccgcagccgaacgaccgagcgcagcgagtcagtgagcgaggaagcaattcgaatccgcgttttctccgtgaaagggaggtgt
cctaggcctctagacgatggggcttttgttatattttactaaatatatattataattaaaaaaaattgaattgtcaatttt
aatgtacacttagttgaaagtgcccctgtccccttggccatatttaacagaagttatttataacgcagctgtttttggagtct
ataaatttataacatcagttactatggatttccctttagttttatggcctaggacgtccccttccccttcgatgctggaggcat
ccttttacgggacaataaataaatttgttgcctcgcctatcggctaacaagttccttcggagtatataaatataggatgttaat
```

```
actgctataaactttagttgcccaatatttatattaggacgccagtggcagtggtaccgccactgcctgcttcgcagtatataa
atataggcagttggcaggcaactgccactgacgtcctattttaatactcccaagtttacttgcctaggcagttggcaggcaaca
aatttatttattgtccactaaaatttatttgcccgaaggggacgtccactaaaatttatttacccgaaggggacgtcctaatat
aaatatggggatgtcaatgctccgttaggaagtaactaacgtttttcaaataaattttatcccggagggaagtaggcagtagcc
cgccactgtcatcctttaagtggatctctcgtcaggcaatttgcttacacctttaaattaaaaattaaatttaaagaaaagtga
gctattaacgcgtctccaatatagtagactttattagaggcagtgtttatataccataaacgtcaaaagtcatttttataactg
gatctcaaaatacctataaacccattgttcttctcttttagctctaagaacaatcaatttataaatatatttattattatgcta
taatataaatactatataaatacatttaccttttataaatacatttaccttttttttaatttgcatgattttaatgcttatgc
tatctttttttatttagtccataaaacctttaaaggaccttttcttatgggatatttatatttcctaacaaagcaatcggcgtc
ataaactttagttgcttacgacgcctgtggacgtcccccccttccccttacgggcaagtaaacttagggattttaatgcaataa
ataaatttgtcctcttcgggcaaatgaattttagtatttaaatatgacaagggtgaaccattactttgttaacaagtgatctt
accactcactattttgttgaattttaaacttatttaaaattctcgagaaagattttaaaaataaactttttaatcttttatt
tatttttttctttttttATGggaagagctactccatggatcctctagagtcgacctgcaggcatgcaagcttgtactcaagctcgt
aacgaaggtcgtgaccttgctcgtgaaggtggcgacgtaattcgttcagcttgtaaatggtctccagaacttgctgctgcatgt
gaagtttggaaagaaattaaattcgaatttgatactattgacaaacttTAAtttttatttttcatgatgtttatgtgaatagca
taaacatcgtttttatttttatggtgtttaggttaaatacctaaacatcattttacattttaaaattaagttctaaagttatc
ttttgtttaaatttgcctgtctttataaattacgatgtgccagaaaaataaaatcttagctttttattatagaatttatcttta
tgtattatattttataagttataataaaagaaatagtaacatactaaagcggatgtaGCGCGTttatcttaacggaaggccagt
ggcagtggcggtgccactgccgaatataaatatggttgagttgcttagtttaccttagcgaaaagaagacttagcagctagcct
taacaaacagttttatattttatgtttgtgttaaataaaaTTAAGAAACTTTAGCTAAAGTTTCCCAACTCATAGAAACGTCAT
CTAAAATTAAAGAACTGTTGTAAATTTCTAAAATGATTAATAAGAATGCTGCAAATAAAAGGATAAATACAGCCATTAAAACAG
TTGTACCCCAGCCTGGTAATACTTTACCTGCTTCTGAGTTAAGTGGACGTAATAAAGTACCTAATGGTGTAACTAAACCAGGTT
CTTGGAAGTCTGAATTTACTTTTGATGGTTTAGCTTTAGAAGTTCCTGTTGCCATaattgattaaatgaattaagcgttattag
cgctatttatttactttctgtaaaaaataaggaaaatattcttcagtgcattccctctcaggattataaatactctgaggata
acgttctctcgtcaaggggttgcttcttgtgagtatagaaacctactagcacaagaaataaattgcataaaaatgtatttacct
aggaccgcagtaggcagtcccttttccccttcagaactgcctgctttaaaagaatgaaaaaactgccttgtctggtaagtaaaa
ctctttaattactcactaaagacgatcttagaagttctttgttcatttttttatttaatataatatttgttatataaaaattaaa
taatttttaattaatgtttaactttgtaaggacagtttcaaagtgacatgaatggctactgcaaaaacgaagtaagttattctt
tctcagggcaaaatttgagtagattaattttgtttaaaaatgtgggacacagtcgtcaagtcttttgaactatctaagagata
tgttgaaaagagaataattttattattaaatgagctatggaaagtccagctttttttctttacctttttttttatggtttcttctg
ttaagtgtaactggctattcagtttatgttagttttggtccaccttcaaaaaaattacgtgatccttttgaagaacacgaagat
taaacaagttaaaaagtactatttttacaagtgacttcggtgcctctgagaaccctagttatagtgatataaaataactagcta
actactttatattttatgaaagtcattttgtcgagcatataaacaaaaacaaaattgctatactaggcagtcacagtgcaact
gtctccgtctccttaaccgagaaagggtaaacgtcttcggtaaagtaacaaactttagttatgttaactgcttgcgagttaacc
atttttttcctccgaaggacaacagttggcagttgccaaactttagtggtctaatatttatattaggcagttggcaggcaact
gcactgacgtcccgaaggggaaggggtttacttacctcctaacggagtatataaatagaataaaatttatttcctgcgctagca
gatttacatactaggattttaatactccgaaggaggcagtggcggtaccactgccactggcgtcctccttccccttcgggcaaa
tgcattttagtgccacttaagtttacttgcctaggcagttggcaggacgtcagtggcagtggtaccgcgactgcctatatttat
atactcctaagtttacttgcctaggcagttggcaggcaactgccactgacgtccttccccttccccttcgggacgtcccttac
gggaatataaatattagtggatatttatatactgcgatgtttacatactccgaaggaggagagctagcagttgcctgccaactg
cctaatataaatattgggcaagtaaacttagaatgtttacatactccgaaggaggacgtcccttacgggaatataaatattagt
ggcagtggtaccgccactgcctccttcggagtattaaaatcctagtatataatataccgtaagggacgtcctccgacggtggca
gtggcggtaccactgccaccggcgtcctaatatacatattgaagtatttaaacctgttagcgcacgctctaacgagtcagtaaa
cttccctttggggcttctaggcagcgcataaatttctaggacgaacgtccactggcgtctcgtaaggagcagtgacaggcca
ctaatgtccccttaatgggtaaataaatggctatcgtctatccatgaagagaccatatattccagtagcaccgttatgatcctc
aaagggtaacaccatttgtatagtattatggtgaaatgcatccctttcagggtagatttatatcttacag
```

1-2404: pUC8 vector (from HindIII [1st base lost during fusion to SphI] to SapI/EcoRI junction.
2405-3199: psbH downstream flanking element (0.80 kb).
3200-3798: atpA promoter/5'UTR (to ATG).
3796-3806: SapI cloning site (nnn/ngaagagc).
3811-3850: NcoI-HindIII polylinker.
3851-4257: rbcL 3' UTR element (stop codon at 3997-3999).
4258-4263: BssHII/MluI join (GCGCGT).
4264-6622: psbH upstream flanking element (2.36 kb) [psbH coding in UPPERCASE 4676-4410].
6622: 1st base of destroyed SphI site.

Region of pASapI around SapI site:

```
                                     M  G  R  A  T  P  W  I  L  -
aactttttaatcttttatttatttttttctttttttATGggaagagctactccatggatcctctagagtcgacctgcaggc
                                         SapI      NcoI BamHI       SalI  PstI SphI
atgcaagcttgtactcaagctcgtaacgaaggtcgtgaccttgctcgtgaaggtggcgacgtaattcgttcagcttgtaa
    HindIII
atggtctccagaacttgctgctgcatgtgaagtttggaaagaaattaaattcgaatttgatactattgacaaacttTAAt
```

2. Construction of the pICS and pAP plasmids

a) Plasmid pICS. The *splB* gene (Genbank accession number WP_094969977) was codon-optimised according to the codon bias found in the *C. reinhardtii* plastome (sequence given below) and cloned into the pASapI vector using *Sap*I and *Sph*I sites flanking the *splB* coding sequence. The resulting plasmid, named pS, contains a unique *Mlu*I restriction site upstream of the *atpA* promoter element that was used for insertion of the next expression cassette (see figure below). The codon-optimized version of *codA* (Genbank accession number CDW51296.1) had already been cloned in a variant of the pASapI vector whereby gene expression is under the control of the *petB* promoter / 5' UTR, instead of that from *atpA*, and the size of the *rbcL* 3'UTR was reduced from 0.4 kb to 0.2 kb (unpublished data). The whole expression cassette was amplified using primers bearing *Mlu*I restriction sites, and was inserted into the pS vector. The resulting vector was named pCS and contained an *Age*I restriction site that was employed to add the third expression cassette. The codon-optimized sequence containing the IBV multi-epitope (Genbank accession number AFJ11183.1) fused to the β subunit of the CTB virus (Genbank accession number AEO36944.1) had been previously cloned into the pSRSapI vector, a modified version of pASapI in which the *atpA* promoter/5'UTR was replaced with that from *psaA-1* (Wannathong et al. 2016). This whole expression cassette (*ibv-ctb*) was amplified with primers bearing the *Age*I restriction site, and then cloned into the pCS vector. The resulting plasmid was named pICS and contained three different coding sequences arranged as three expression cassettes.

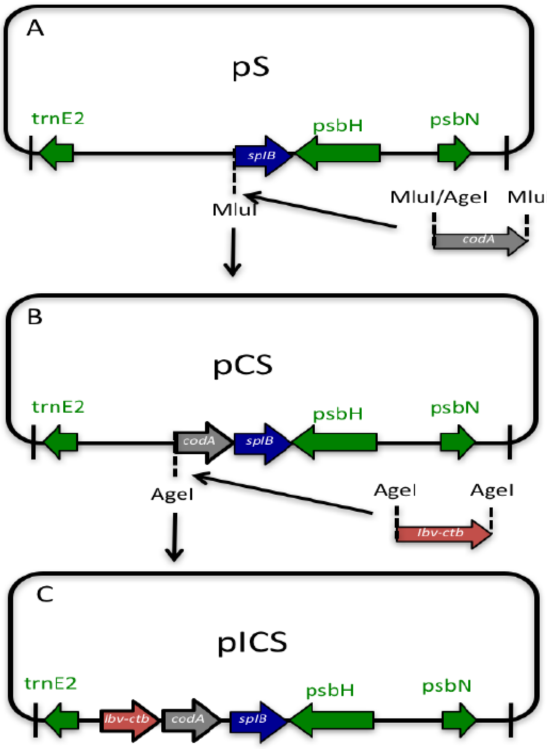

b) Plasmid pAP: The target sequences for the *chlL* gene were amplified from genomic DNA of the cell wall-less strain cw-15; the obtained product was cloned into the pJet vector, and the resulting plasmid was named pchlL, it was subsequently digested with *Bst*BI and *Nde*I restriction enzymes to allow the insertion of the aminoglycoside-3-adenyltransferase (*aadA*) expression cassette, which confers

resistance to spectinomycin (Goldschmidt-Clermont, 1991). The expression cassette was amplified using primers bearing the *Ac1*I and *Nde*I restriction sites. The product was inserted into the pchlL plasmid and the resulting construct was named pA. Subsequently, this plasmid was digested with the *Xma*I restriction enzyme, which permitted the incorporation of the expression cassette containing the *cpl-1* gene, which was amplified using primers carrying the *Xma*I restriction site from the pSRSapI vector (Stoffels et al., 2017), the resulting amplicon was cloned into the pAchlL plasmid to yield the pAP construct, which was used to transform the ICS3 strain of *C. reinhardtii*.

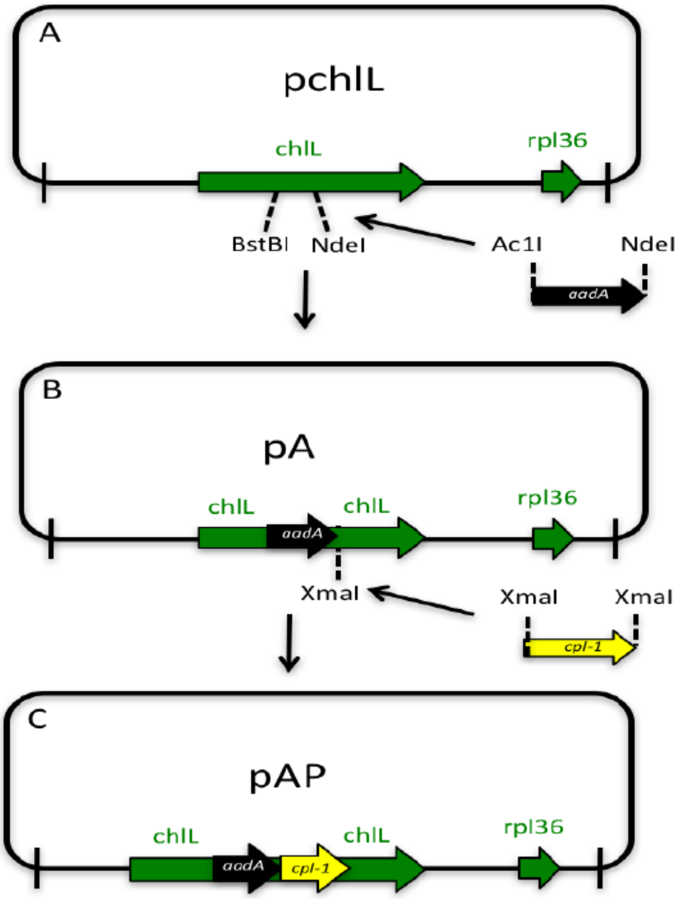

3. Sequences of key DNA elements

***splB* CDS**

ATGAACAAAAACGTAGTTATTAAATCATTAGCTACTTTAACAATTTTAACTTCTGTTACTGGTATTGG
TACTACTTTAGTTGAAGAAGTACAACAAACAGCTAAAGCTGAAAACAACGTTACTAAAATTCAAGATA
CTAACATTTTCCCTTATACTGGTGTTGTAGCTTTCAAATCAGCTACTGGTTTCGTTGTAGGTAAAAAC
ACTATTTTAACAAACAAACACGTTTCTAAAAACTACAAAGTAGGTGACCGTATTACTGCTCACCCAAA
CTCTGACAAAGGTAACGGTGGTATTTACAGTATTAAAAAAATTATTAACTACCCAGGTAAAGAAGATG
TTTCTGTTATTCAAGTTGAAGAACGTGCTATTGAACGTGGTCCAAAAGGTTTCAACTTCAACGATAAC
GTTACTCCATTCAAATATGCTGCTGGTGCTAAAGCTGGTGAACGTATTAAAGTTATTGGTTACCCACA
CCCATACAAAAACAAATATGTTTTATATGAATCAACTGGTCCAGTAATGTCTGTTGAAGGTTCATCAA
TTGTATACTCAGCTCACACAGAATCAGGTAACTCAGGTTCTCCAGTTTTAAACTCTAACAACGAATTA
GTTGGTATTCACTTCGCTTCTGATGTTAAAAACGATGATAACCGTAACGCTTACGGTGTTTATTTCAC

TCCAGAAATTAAAAAATTCATTGCTGAAAACATTGATAAATACCCATACGATGTTCCAGATTACGCTT
AA

***codA* CDS**
ATGTCTAACAACGCTTTACAAACAATTATTAACGCTCGTTTACCAGGTGAAGAAGGTTTATGGCAAAT
TCACTTACAAGACGGTAAAATTTCAGCTATTGATGCTCAATCTGGTGTAATGCCAATTACTGAAAACT
CTTTAGATGCTGAACAAGGTTTAGTTATTCCACCATTCGTTGAACCACACATTCACTTAGATACTACA
CAAACAGCTGGTCAACCAAACTGGAACCAATCAGGTACTTTATTTGAAGGTATTGAGCGTTGGGCTGA
ACGTAAAGCTTTATTAACACACGACGACGTTAAACAACGTGCTTGGCAAACATTAAAATGGCAAATTG
CTAACGGTATTCAACACGTACGTACTCACGTAGACGTTTCTGATGCTACTTTAACAGCTTTAAAAGCT
ATGTTAGAAGTTAAACAAGAAGTAGCTCCATGGATTGACTTACAAATTGCTGCTTTCCCACAAGAAGG
TATTTTATCATACCCAAACGGTGAAGCTTTATTAGAAGAAGCTTTACGTTTAGGTGCTGATGTTGTTG
GTGCTATTCCACACTTCGAATTTACACGTGAATATGGTGTTGAATCTTTACACAAAACATTTGCTTTA
GCTCAAAAATATGATCGTTTAATTGATGTTCACTGTGACGAAATTGATGACGAACAATCACGTTTCGT
TGAAACAGTAGCTGCTTTAGCTCACCACGAAGGTATGGGTGCTCGTGTTACTGCTTCACACACTACAG
CTATGCACTCTTACAACGGTGCTTACACTTCTCGTTTATTCCGTTTATTAAAAATGTCTGGTATTAAC
TTCGTTGCTAACCCATTAGTAAACATTCACTTACAAGGTCGTTTCGATACTTACCCAAAACGTCGTGG
TATTACACGTGTTAAAGAAATGTTAGAATCAGGTATTAATGTTTGTTTTGGTCACGACGACGTTTGTG
GTCCTTGGTACCCTTTAGGTACTGCTAACATGTTACAAGTTTTACACATGGGTTTACACGTATGTCAA
TTAATGGGTTACGGTCAAATTAACGACGGTTTAAACTTAATTACTCACCACTCTGCTCGTACTTTAAA
CTTACAAGACTACGGTATTGCTGCTGGTAACTCAGCTAACTTAATTATTTTACCAGCTGAAAACGGTT
TCGATGCTTTACGTCGTCAAGTTCCAGTACGTTACTCAGTTCGTGGTGGTAAAGTTATTGCTTCAACT
CAACCAGCTCAAACAACTGTTTATTTAGAACAACCAGAAGCTATTGACTACAAACGTTACCCATACGA
TGTTCCAGATTACGCTTAA

***ibv-ctb* CDS**
ATGTCAACTGGTCAAAACTTAACAAGTTGTCCATACGTTTCTTACGGTCGTTTCTGTATTGAACCAGA
TGGTTCATTAGCTGCTTACCAACACGGTTACTGGCGTCGTCAACACCGTTTCAAACCATCTAAAGGTG
GTGGTTCAGGTGGTTCAAAATTACAACCAGATGGTTTACACTTAAAATTCGAATTTACTACAGTAGTT
TCTCGTGATGATCCACAATTCGATAACTACGTTAAAATTTGTGACGAATGTGTAGATGGTGTAGGTAC
TCGTCCAAAAGATGAAGTAGTTCGTCCAAAATCACGTTCTTCATCACGTCCAGCTACTCGTGGTAACT
CTCCAGCTCCACGTCAACAACGTCAAAAAAAAGAAAAAAAACCAAAAAAACAAGAAGATGATGTTGAT
AAAGCTTTAACATCAGATGAAGAACGTAACAACGCTCAATTAGAATTCGATGATGAACCAAAAGTTAT
TAACTGGGGTGATTCTGCTTTAGGTGAAAACGAATTAATTTGGATCCTTGGTGGTGGTGGTTCTTCAG
GTGGTGGTGGTGGTGGTTCTATGACACCACAAAATATTACTGATTTATGTGCAGAATATCACAATACA
CAAATTCACACATTAAATGATAAAATTTTTTCATATACTGAATCATTAGCTGGTAAACGTGAAATGGC
TATTATTACATTCAAAAACGGTGCTACATTTCAAGTAGAAGTTCCTGGTTCACAACACATTGATTCTC
AAAAAAAAGCTATTGAACGTATGAAAGATACATTACGTATTGCTTATTTAACAGAAGCTAAAGTAGAA
AAATTATGTGTTTGGAATAATAAAACTCCTCATGCAATTGCAGCTATTTCAATGGCAAATTACCCATA
CGATGTTCCAGATTACGCTTAA

***aadA* CDS**
ATGGCTCGTGAAGCGGTTATCGCCGAAGTATCAACTCAACTATCAGAGGTAGTTGGCGTCATCGAGCG
CCATCTCGAACCGACGTTGCTGGCCGTACATTTGTACGGCTCCGCAGTGGATGGCGGCCTGAAGCCAC
ACAGTGATATTGATTTGCTGGTTACGGTGACCGTAAGGCTTGATGAAACAACGCGGCGAGCTTTGATC
AACGACCTTTTGGAAACTTCGGCTTCCCCTGGAGAGAGCGAGATTCTCCGCGCTGTAGAAGTCACCAT
TGTTGTGCACGACGACATCATTCCGTGGCGTTATCCAGCTAAGCGCGAACTGCAATTTGGAGAATGGC
AGCGCAATGACATTCTTGCAGGTATCTTCGAGCCAGCCACGATCGACATTGATCTGGCTATCTTGCTG
ACAAAAGCAAGAGAACATAGCGTTGCCTTGGTAGGTCCAGCGGCGGAGGAACTCTTTGATCCGGTTCC
TGAACAGGATCTATTTGAGGCGCTAAATGAAACCTTAACGCTATGGAACTCGCCGCCCGACTGGGCTG
GCGATGAGCGAAATGTAGTGCTTACGTTGTCCCGCATTTGGTACAGCGCAGTAACCGGCAAAATCGCG
CCGAAGGATGTCGCTGCCGACTGGGCAATGGAGCGCCTGCCGGCCCAGTATCAGCCCGTCATACTTGA
AGCTAGACAGGCTTATCTTGGACAAGAAGAAGATCGCTTGGCCTCGCGCGCAGATCAGTTGGAAGAAT
TTGTCCACTACGTGAAAGGCGAGATCACTAAGGTAGTTGGCAAATAA

***cpl-1* CDS**

ATGGTTAAAAAAAATGATTTATTCGTTGATGTTTCATCACACAATGGTTATGATATTACAGGTATTTT
AGAACAAATGGGTACTACAAATACAATTATTAAAATTTCAGAATCAACAACATATTTAAATCCATGTT
TATCAGCTCAAGTTGAACAATCAAATCCAATTGGTTTTTATCACTTTGCTCGTTTTGGTGGTGATGTT
GCTGAAGCTGAACGTGAAGCTCAATTTTTTTTAGATAATGTTCCAATGCAAGTTAAATATTTAGTTTT
AGATTATGAAGATGATCCATCAGGTGATGCTCAAGCTAATACAAATGCTTGTTTACGTTTTATGCAAA
TGATTGCTGATGCTGGTTATAAACCAATTTATTATTCATATAAACCATTTACACACGATAATGTTGAT
TATCAACAAATTTTAGCTCAATTTCCAAATTCATTATGGATTGCTGGTTATGGTTTAAATGATGGTAC
TGCTAATTTTGAATATTTTCCATCAATGGATGGTATTCGTTGGTGGCAATATTCATCAAATCCATTCG
ATAAAAATATTGTTTTATTAGATGATGAAGAAGATGATAAACCAAAAACAGCTGGTACATGGAAACAA
GATTCAAAAGGTTGGTGGTTTCGTCGTAATAATGGTTCATTTCCATATAATAAATGGGAAAAAATTGG
TGGTGTTTGGTATTATTTCGATTCTAAAGGTTATTGTTTAACATCAGAATGGTTAAAAGATAATGAAA
AATGGTATTATTTAAAAGATAATGGTGCTATGGCTACAGGTTGGGTTTTAGTTGGTTCAGAATGGTAT
TATATGGATGATTCAGGTGCTATGGTAACTGGTTGGGTAAAATATAAAAATAATTGGTATTATATGAC
TAATGAACGTGGTAATATGGTTTCAAATGAATTTATTAAATCAGGTAAAGGTTGGTATTTTATGAATA
CAAATGGTGAATTAGCAGATAATCCTTCATTTACAAAAGAACCAGATGGTTTAATTACAGTTGCTTAT
CCATATGATGTTCCAGATTATGCTTAA

***atpA* promoter/5′UTR**

CGCGTCTCCAATATAGTAGACTTTATTAGAGGCAGTGTTTATATACCATAAACGTCAAAAGTCATTTT
TATAACTGGATCTCAAAATACCTATAAACCCATTGTTCTTCTCTTTTAGCTCTAAGAACAATCAATTT
ATAAATATATTTATTATTATGCTATAATATAAATACTATATAAATACATTTACCTTTTTATAAATACA
TTTACCTTTTTTTTAATTTGCATGATTTTAATGCTTATGCTATCTTTTTTATTTAGTCCATAAAACCT
TTAAAGGACCTTTTCTTATGGGATATTTATATTTTCCTAACAAAGCAATCGGCGTCATAAACTTTAGT
TGCTTACGACGCCTGTGGACGTCCCCCCCTTCCCCTTACGGGCAAGTAAACTTAGGGATTTTAATGCA
ATAAATAAATTTGTCCTCTTCGGGCAAATGAATTTTAGTATTTAAATATGACAAGGGTGAACCATTAC
TTTTGTTAACAAGTGATCTTACCACTCACTATTTTTGTTGAATTTTAAACTTATTTAAAATTCTCGAG
AAAGATTTTAAAAATAAACTTTTTTAATCTTTTATTTATTTTTTCTTTTTT[ATG]

***psaA-1* promoter/5′UTR**

AAGCTTTCTTAATTCAACATTTTTAAGTAAATACTGTTTAATGTTATACTTTTACGAATACACATATG
GTAAAAAATAAAACAATATCTTTAAAATAAGTAAAAATAATTTGTAAACCAATAAAAAATATATTTAT
GGTATAATATAACATATGATGTAAAAAAAACTATTTGTCTAATTTAATAACCATGCATTTTTTATGAA
CACATAATAATTAAAAGCGTTGCTAATGGTGTAAATAATGTATTTATTAAATTAAATAATTGTTATTA
TAAGGAGAAATCC[ATG]

***petB* promoter/5′UTR**

ATTGTACCACAATAAATAAATTTGTCCTCCTTCGGAGTATGTAAACCCCTTCGGGCAACTAAAGTTTA
TGCTAAGTTTACTTGCCCTATATTTATATACCCGAAGGGGAAGGGGAAGGAGGCAGCCCCAGAGGGGA
CATTTATGTAAAAAGTTACTTTGGAGTATAGAAATATGGTAAGAGGTGACATTCTTATATTTAATACA
TAATAGTTCAATTGTATAATATTAAAACTTTTGAATTACTAACGTTTTTTTTATTTTATATATAATAT
ATATTATAACTTTAATTTAAACTTTAAATAATTAAGCTACACATTTTTTTAGTCTTAAGAAAGCCTAA
TGGTCATGTCACAATCTTATAAAAATTTTAT[ATG]

***rbcL* 3′UTR (long version including part of coding region [underlined])**

GTACTCAAGCTCGTAACGAAGGTCGTGACCTTGCTCGTGAAGGTGGCGACGTAATTCGTTCAGCTTGT
AAATGGTCTCCAGAACTTGCTGCTGCATGTGAAGTTTGGAAAGAAATTAAATTCGAATTTGATACTAT
TGACAAACTTTAATTTTTATTTTTCATGATGTTTATGTGAATAGCATAAACATCGTTTTTATTTTTAT
GGTGTTTAGGTTAAATACCTAAACATCATTTTACATTTTTAAAATTAAGTTCTAAAGTTATCTTTTGT
TTAAATTTGCCTGTCTTTATAAATTACGATGTGCCAGAAAAATAAAATCTTAGCTTTTTATTATAGAA
TTTATCTTTATGTATTATATTTTATAAGTTATAATAAAAGAAATAGTAACATACTAAAGCGGATGTA

***rbcL* 3′UTR (short version)**

TTTTTATTTTTCATGATGTTTATGTGAATAGCATAAACATCGTTTTTATTTTTATGGTGTTTAGGTTA
AATACCTAAACATCATTTTACATTTTTAAAATTAAGTTCTAAAGTTATCTTTTGTTTAAATTTGCCTG
TCTTTATAAATTACGATGTGCCAGAAAAATAAAATCTTAGCTTTTTATTATAGAATTTATCTTTATGT
ATTATATTTTATAAGTTATAATAAAAGAAATAGTAACATACTAAAGCGGATGTA

Fig. S1 Comparison of mixotrophic growth rates of the transformant lines

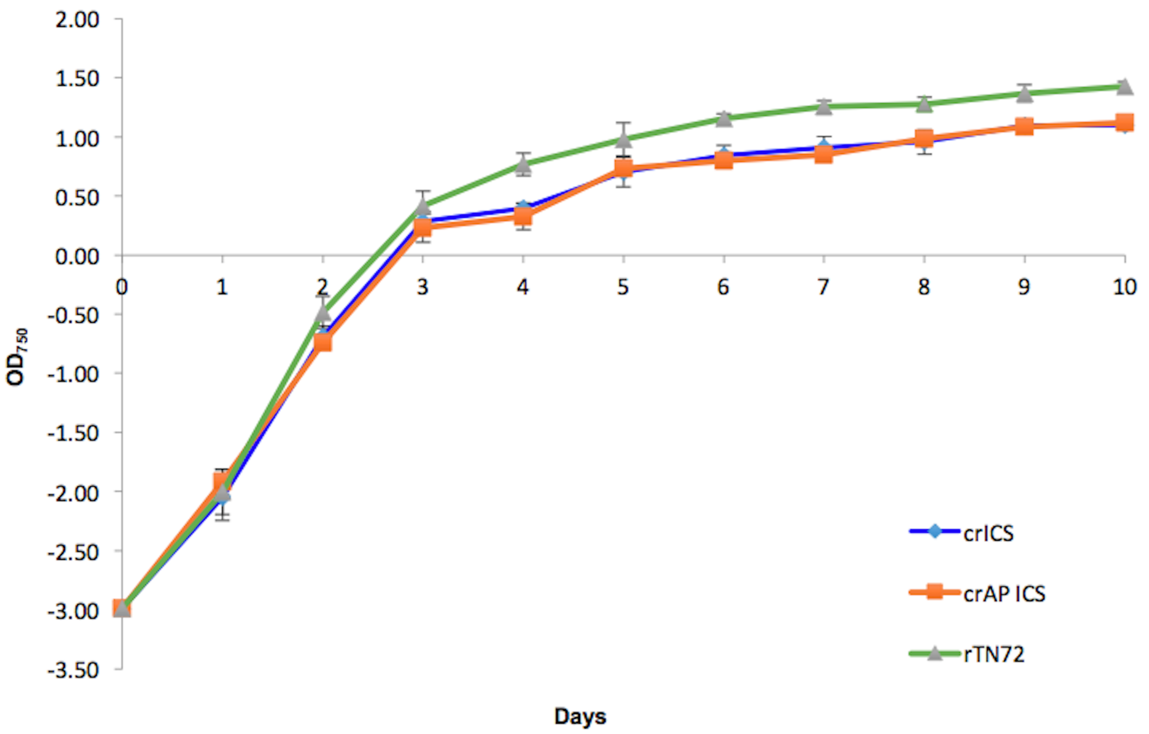

Strain **ICS3** (expressing *ibv-ctb*, *codA* and *splB*: hence four copies of *rbcL* 3'UTR); **AP_ICS1** (expressing *ibv-ctb*, *aadA* and *cpl-1*: four copies of *rbcL* 3'UTR) and **rTN72** (a control transformant generated using pASapI so devoid of any transgenes: two copies of *rbcL* 3'UTR) were grown under identical conditions in acetate-containing TAP medium in the light. Cell density was assessed by daily measurement of optical density at 750 nm. The data presented are averages of three independent measurements. *Note*: acetate becomes exhausted at the end of the exponential phase so the difference seen as the cultures enter stationary phase is principally due to differences in phototrophic performance.

References

Goldschmidt-Clermont M. Transgenic expression of aminoglycoside adenine transferase in the chloroplast: a selectable marker of site-directed transformation of chlamydomonas. *Nucleic Acids Res* 1991;19:4083–4089. doi: 10.1093/nar/19.15.4083

Stoffels L, Taunt HN, Charalambous B, Purton S. Synthesis of bacteriophage lytic proteins against *Streptococcus pneumoniae* in the chloroplast of *Chlamydomonas reinhardtii*. *Plant Biotechnol J* 2017:15;1130–1140. doi: 10.1111/pbi.12703

Wannathong T, Waterhouse JC, Young REB, Economou CK, Purton S. New tools for chloroplast genetic engineering allow the synthesis of human growth hormone in the green alga *Chlamydomonas reinhardtii*. *Appl Microbiol Biotechnol* 2016;100:5467–5477. doi:10.1007/s00253-016-7354-6
